# Comparison of Appetite-regulating Hormones and Body Composition in Pediatric Patients in Predialysis Stage of Chronic Kidney Disease and Healthy Control Group

**Published:** 2015-01

**Authors:** Mohammad Hassan Eftekhari, Maryam Ranjbar-Zahedani, Mitra Basiratnia, Abbas Rezaianzadeh, Shiva Faghih

**Affiliations:** 1 Department of Nutrition, School of Nutrition and Food Sciences, Shiraz University of Medical Sciences, Shiraz, Iran;; 2 Department of Nephrology Urology Research Center, Shiraz University of Medical Sciences, Shiraz, Iran;; 3 Department of Epidemiology, Research Center for Health Sciences, Shiraz University of Medical Sciences, Shiraz, Iran

**Keywords:** Chronic renal insufficiency, Ghrelin, Leptin, Obestatin

## Abstract

**Background:**

Protein-energy malnutrition (PEM) is a common complication in pediatric patients with chronic kidney disease (CKD). Components incorporated in the regulation of appetite and body composition appear to be of the focus in renal insufficiency and may influence the CKD-associated PEM. The purpose of this study was to investigate plasma levels of appetite-regulating hormones and their correlation with the body composition variables in a pediatric in predialysis stage of CKD.

**Methods:**

Thirty children with CKD in predialysis stage were selected and compared with 30 healthy sex- and age-matched controls. Blood samples were collected in fasting. Serum total ghrelin, leptin, and obestatin levels were measured using enzyme immunometric assay methods. Anthropometric parameters measurement and body composition analysis were done using the bioelectric impedance analysis (BIA) method.

**Results:**

Patients showed insignificant elevated total ghrelin (105.40±30.83 ng/l), leptin (5.32±1.17 ng/ml) and obestatin (5.07±1.09 ng/ml) levels in comparison with healthy participants. By using BIA, patients had significantly different Dry Lean Weight (P=0.048), Extra Cellular Water (P=0.045), Body Cell Mass (BCM) (P=0.021), Basal Metabolic Rate (P=0.033) and Body Mass Index (P=0.029) compared with controls. Furthermore, the total body water was slightly and the ECW was significantly higher in CKD participants. There were significant negative correlation between obestatin and BCM (r=**-**0.40, P=0.03) and fat free mass index (FFMI) (r=**-**0.40, P=0.029) in patients.

**Conclusion:**

It seems that our results are insufficient to clarify the role of appetite-regulating hormones in PEM in CKD patients. It is apparent that there are still many unknown parameters related to both appetite regulating and CKD-associated PEM.

## Introduction

Protein energy malnutrition (PEM) and growth retardation are common complications in pediatric patients and strong predictors of morbidity and mortality.^1^^-^^3^ The progressive prevalence of PEM, a condition of loss of both the muscle and visceral protein stores, arises along with the loss of residual renal function in chronic kidney disease (CKD) patients.^4^ Restrictions in some food groups, increase of the catabolism due to inflammatory cytokines, low appetite, uremic toxins and metabolic acidosis as well as a decrease in anabolic hormones, contribute to malnutrition in such patients.^5^ Recently, alteration in appetite-regulating hormones is considered as an additional probable cause of PEM. An interruption between the balance in anorexigenic and orexigenic hormones may influence the progression of CKD-associated protein energy malnutrition in pediatric patients.^6^

Ghrelin, which is predominantly produced by the PD-1 cells of the stomach, increases food intake and induces obesity, acts as leptin antagonist. It also stimulates neuropeptide Y (NPY) and agouti related peptide (AgPR) in the arcuate nucleus of hypothalamus and inhibit the reduction of food intake, which is mediated by leptin. Ghrelin has additional growth hormone-releasing properties and binds to the growth hormone secretagogue receptor. Furthermore, ghrelin has a positive inotropic effect on the cardiovascular system and decline blood pressure. Besides, it can block the production of anorectic proinflammatory cytokines, including IL-1β, IL-6 and TNF-α. The combination of these actions suggests that, this peptide has a beneficial effect on the prevention of cachexia and PEM.^6^^-^^12^ Ghrelin administration can improve appetite in adult patients on peritoneal dialysis.^13^^,^^14^ Recent studies showed that, the concentration of plasma ghrelin is inversely correlated with body mass index (BMI) and age in healthy children.^6^ Few number of anorexigenic peptide which compete against ghrelin on appetite regulation are leptin and obestatin. Leptin is mainly produced by adipocytes and acts via the particular receptor OB-Rb, decreases food intake by motivation the anorexigenic peptide alpha-melanocyte-stimulating hormone peptide (α-MSH). It suppresses the orexigenic neuropeptide Y (NPY) and AgRP in the arcuate nucleus of the hypothalamus.^6^^,^^7^

Obestatin or ghrelin-associated peptide, which is originated from the same ghrelin gene and produced by the stomach, has an opposite effect on feeding and anorexigenic properties.^15^

Investigation on appetite regulatory hormones in CKD patients has recently attracted a lot of attention due to their effect on appetite reduction and weight loss and consequently PEM. Previous studies showed that, in pediatrics CKD patients, renal insufficiency led to the accumulation of the ghrelin and anorexigenic hormones leptin and obestatin.^6^ Up to now, there has been no study investigating the alteration of serum appetite regulatory hormones and their relation to nutritional status of the Iranian children with renal insufficiency. Consequently, the aim of the present study was to determine the plasma ghrelin, leptin and obestatin concentrations in pediatric patients in predialysis stage of CKD, and their relations with body compositions.

## Patients and Methods

The sample size was calculated based on the leptin value in Büscher et al. study,^6^ regarding power of 90% and α=5%. In this case-control study, 30 patients (22 boys and 8 girls) were compared with 30 healthy individuals as controls.

Key inclusion criteria included age range of 6 to 20 years old and GFR range=15-50 ml/min/1.73 m^2^. Patients were not on dialysis and did not receive kidney transplantation or growth hormone therapy. All participants were recruited from the Pediatric Nephrology Clinic of Shiraz University of Medical Sciences.

The major causes of CKD were reported to be obstructive uropathy, congenital anomalies of kidney or urinary tract and hemolytic uremic syndrome. Bicarbonate, multivitamin-minerals, anti-hypertensive drugs, erythropoietin, oral iron, and phosphate binders were the drugs that had been taken by most of the patients as a conservative treatment. Thirty healthy age- and sex-matched children served as a control group. They visited pediatrics physicians for the treatment of minor diseases that did not impair renal function, and had no metabolic or endocrinological disorders. The control group did not receive any regular medication and had no history of chronic diseases. Written consent was obtained from their caregivers and all assessments were solely done by the researcher. The study was approved by the Ethical Committee of Shiraz University of Medical Sciences.

*Anthropometric and Body Composition Assessment*

Bioelectric impedance analysis (BIA) as a simple and convenient method in measuring body composition has attracted the interest of dietitians and nephrologists for the evaluation of the nutritional status in CKD patients. It can provide information on both hydration status and body composition.^16^^,^^17^ Anthropometric measurements were performed early in the afternoon, weight was measured to the nearest 0.1 kg with minimum clothing and height was measured to the nearest 0.5 cm while patients were barefooted, with their legs together in an upright position. Body mass index (BMI) was calculated as the ratio of weight/hight^2^ (kg/m^2^). BIA was used to determine body components such as body fat, Dry Lean Weight (DLW), Total Body Water (TBW), Extra Cellular Water (ECW), Intra Cellular Water (ICW), Body Cell Mass (BCM), Basal Metabolic Rate (BMR), Body Fat Mass Index (BFMI) and Fat Free Mass Index (FFMI). It was used in the post absorptive state, by injecting 800 microampere and 50 kHz alternating sinusoidal current with a standard tetra polar technique (Bodystat QuadScan 4000 device, England). BIA was performed in standard conditions; a quiet environment, ambient temperature of 22-24°C, after voiding and being at least 20 minutes at rest in the supine position and without consumption of coffee or tea prior to the measuring.

*Biochemical Assessment*

Blood samples were collected from all participants after at least 8-hour fasting in a standard manner. Serum was separated and frozen immediately at -70ºC until being analyzed. The levels of ghrelin, leptin, and obestatin peptide hormones were measured using the enzyme immunometric assay method (GLORY science, IBL international and GLORY science kit respectively for ghrelin, leptin, and obestatin). The acceptable normal values for these parameters were considered to be 100-3000 ng/l, 1-100 ng/ml and 0.05-10 ng/ml for ghrelin, leptin and obestatin respectively.

*Statistical Analysis*

The normality of distribution was checked for all variables by Kolmogorov-Smirov one-sample test. Data processing and analysis were done with SPSS software version 16 for Windows (SPSS Inc., Chicago, USA). Normally distributed variables were expressed as mean±SD and were compared by Independent Student’s *t* test. Pearson’s correlation coefficient was used to examine the relation between variables. Significance was set at P<0.05.

## Results

The demographic characteristics of CKD and healthy children are shown in table 1. As shown, mean weight and mean BMI of CKD children were significantly lower than the control group and the height was slightly different between the two groups of the study.

**Table 1:** Demographic characteristics of patients with CKD and control participants

** **	**Patients 30 (22 boys and 8 girls)**	**Control 30 (22 boys and 8 girls)**	**P value‡**
Age (year)	12.1±4.03†	12.27±4.39	>0.05
Height (cm)	135±20.44	145±24.19	0.074
Weight (kg)	31.73±15.32	43.58±22.1	0.020
BMI1 (kg/m2)	16.39±3.61	18.86±4.83	0.029

†mean±SD; ‡Independent Sample *t* test; 1: BMI, body mass index

Table 2 shows the nutritional status of the study groups based on BMI.^18^ Based on BMI, almost 30% and 16.7% of CKD-patients and controls were categorized as underweight, respectively.

**Table 2:** Comparison of the nutritional status of the study groups based on BMI

** **	**Underweight ** **(BMI percentile<5%)**	**Normal weight ** **(BMI percentile=5-85%)**	**Over weight ** **(BMI percentile=85-95%)**	**Obese ** **(BMI percentile>95%)**
Patients	30.00†/9‡	63.30/19	6.70/2	0.00/0
Controls	16.70/5	53.30/16	13.30/4	16.70/5

†percent/‡number

Comparison of the biochemical indices is shown in table 3. As shown, there was no significant difference between ghrelin, leptin, and obestatin in patients compared with the healthy participants.

**Table 3:** Biochemical parameters in CKD patients and control participants

** **	**Patients**	**Control**	**P value‡**
Ghrelin (ng/l)	105.40±30.83†	101.93±29.06	0.65
Leptin (ng/ml)	5.32±1.17	4.90±1.006	0.14
Obestatin (ng/ml)	5.07±1.09	4.55±1.003	0.06

†mean±SD; ‡Independent Sample *t* test

The results of BIA are shown in table 4. There were no significant differences between the mean values of body fat, lean body mass, TBW, ICW, BFMI and FFMI, but the mean values of DLW (P=0.048), ECW (P=0.045), BCM (P=0.021) and BMR were significantly different between uremic patients and healthy control participants.

**Table 4:** Body composition variables in CKD patients and control participants

** **	**Patients**	**Control**	**P value‡**
Body fat (%)	18.32±9.24†	21.13±8.72	0.23
Lean body weight (%)	81.36±9.80	78.87±8.72	0.30
DLW (kg)	6.37±3.66	8.51±4.44	0.048
TBW (%)	61.84±6.88	59.37±6.67	0.16
ECW (%)	36.11±7.15	31.98±8.27	0.045
ICW (%)	42.29±10.65	39.20±11.39	0.28
BCM (kg)	17.32±5.94	21.71±8.13	0.021
BMR (kcal/day)	1155±288	1354±402	0.033
BFMI (kg/m2)	3.07±1.97	4.25±2.64	0.057
FFMI (kg/m2)	13.28±2.97	14.61±2.99	0.093

DLW: Dry lean weight; TBW: Total body water; ECW: Extra cellular water; ICW: Intra cellular water; BCM: Body cell mass; BMR: Basal metabolic rate; BFMI: Body fat mass index; FFMI: Fat free mass index; †mean±SD; ‡Independent Sample *t* test

**Figure 1:**
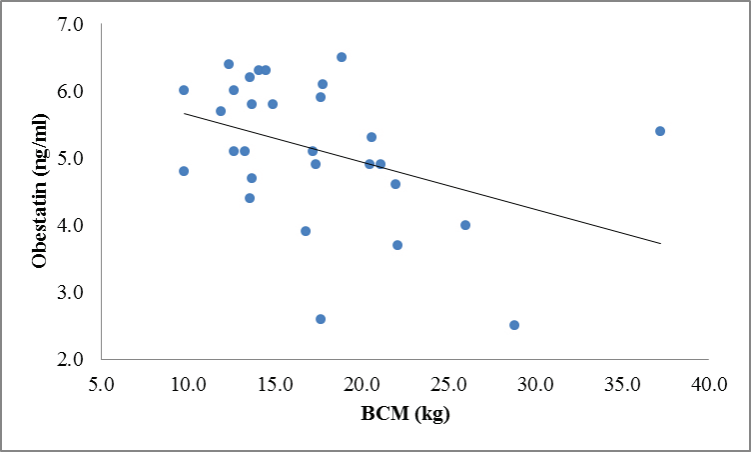
Correlation between obestatin and BCM levels in patients (r=-0.40, P=0.03).

**Figure 2:**
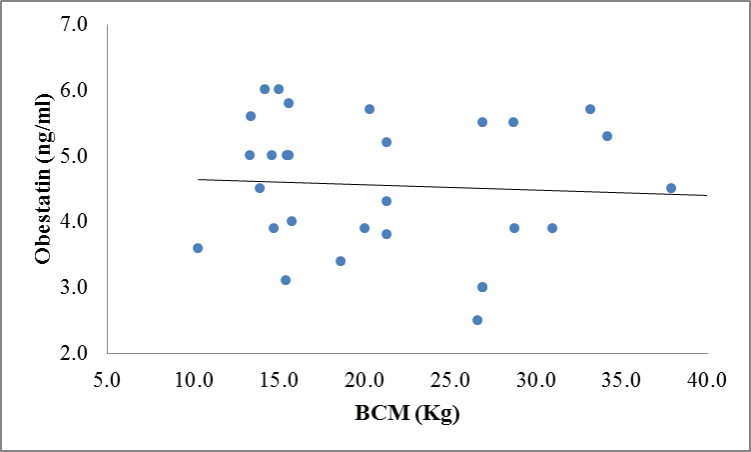
Correlation between obestatin and BCM levels in controls (r=-0.063, P=0.739).

**Figure 3:**
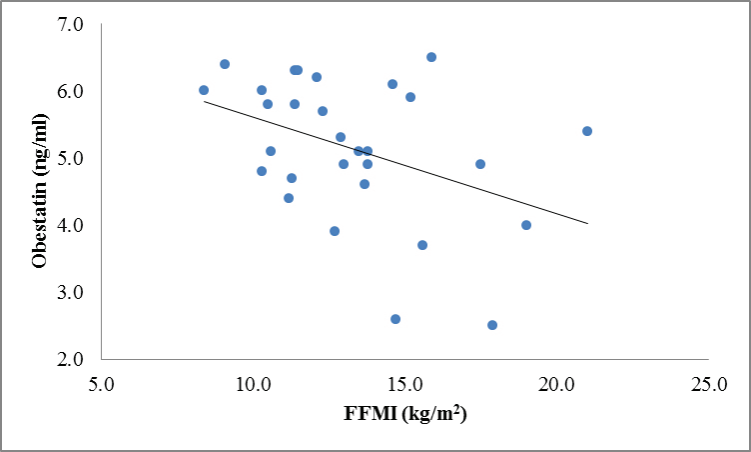
Correlation between obestatin and FFMI levels in patients (r=-0.40, P=0.029).

**Figure 4:**
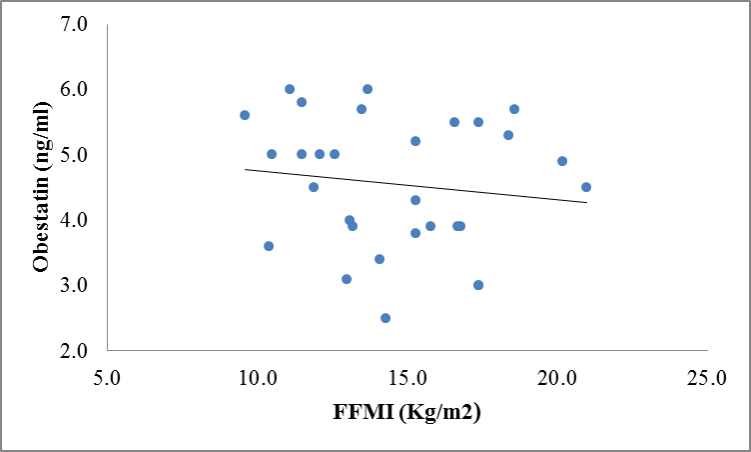
Correlation between obestatin and FFMI levels in controls (r=-0.132, P=0.486).

There were no correlation between ghrelin, leptin and obestatin hormones and body composition variables except for significant inverse correlation between obestatin and BCM (r=**-**0.40, P=0.03) and FFMI(r=-0.40, P=0.029) in CKD patients (figure 1 and 3 in comparison with figure 2 and 4 in the controls).

## Discussion

Protein-energy malnutrition, due to its effect on the quality of life as well as impaired normal growth, is a predominant problem in uremic pediatric patients.^1^^-^^3^ An imbalance between an orexigenic and anorexigenic hormone, probably amplify the CKD-associated malnutrition in pediatric patients.^6^^,^^7^ Therefore, we directed our investigation to obtain more information about the regulation of some appetite regulating hormones and their correlation with body composition indices in Iranian pediatric CKD patients.

Previous studies showed the elevation of total plasma ghrelin levels in pediatric with renal insufficiency and children undergoing dialysis compared with healthy control and renal transplant recipients (RTx).^6^ It was expected to find higher ghrelin levels in children with CKD; however, a similar ghrelin levels in CKD patients and in the control participants were found. Although any association between ghrelin and PEM in early stage of CKD in children could not be found, there might be insensitivity to ghrelin in pediatric with CKD due to uremia or other metabolic disorders.

Patients with moderate renal insufficiency in the predialysis stage of CKD participated in this study. Clinically, these patients suffered from PEM that had already led to a lower BMI, BCM, and DLW compared with healthy participants. Even though the total plasma ghrelin levels were similar in our patients compared with healthy participants, poor appetite also was seen in most of the patients. This situation might be due to “ghrelin resistance” which was mentioned in previous researches. Many studies reported that plasma ghrelin levels were elevated in cachectic patients because of different underlying disorders. This phenomenon has been called “ghrelin resistance”, which might be a compensatory response reflecting imbalanced energy state.^14^ On the other hand, in the present study, we only measured total ghrelin levels, while after that ghrelin has been synthesized in stomach cells. For binding to its receptors, it needs post translational modification by adding an octanoyl group to serin-3 (acylation). In fact, acylated ghrelin stimulates food intake and des-acyl form has anorexigenic effect.^6^ In those studies that measured the acylated moiety of ghrelin, elevation in both ghrelin and des-acyl and alteration of the acyl ghrelin/total ghrelin ratio were reported in the end stage of renal disease patients undergoing dialysis in comparison with controls, in pediatric and adults patients, respectively.^6^^,^^15^ Des-acyl ghrelin levels have been linked with malnutrition in human^19^^,^^20^ and elevated levels of des-acyl ghrelin may contribute to appetite decrease in uremic patients.

Leptin, as another anorexigenic peptide block the ghrelin activity as well as stimulating of proopiomelanocortin producing neurons in the arcuate nucleus of hypothalamus.^6^^,^^7^ In our study, although the difference was not significant, serum concentration levels of leptin were higher in the patients in the predialysis stage in comparison with the healthy participants. Enhanced leptin levels can be another powerful hypothesis explaining reduced appetite under these situations. In Büscher et al. study, leptin serum levels were significantly elevated in RTx patients compared with control and uremic patients.^6^ Generally in the literature, data on leptin regulation during uremia are inconsistence, showing elevated or unchanged leptin in CKD patients.^21^^-^^23^ Previous studies showed that leptin positively correlated with BMI^6^, but we did not observe this correlation in our study. This might be due to the small sample size or slight change of leptin that contributed to the moderate renal insufficiency of our patients.

Obestatin has an opposite impact on appetite.^15^ We found that plasma obestatin levels increased slightly in pediatric patients with renal insufficiency. Our data were somehow in agreement with the result of previous studies, which showed an elevated obestatin level in pediatric and adult on hemodialysis.^6^^,^^15^ Elevation in obestatin levels might be due to its reduced renal clearance and can intensify the appetite-inhibiting effect of elevated leptin level and “ghrelin resistance”.

We also analyzed our data regarding appetite-regulating hormones in relation to body composition variables, which were measured by BIA method. However, we did not notice any significant correlation among these variables except for a significant negative correlation between obestatin, BCM, and FFMI in patients. This is possibly because of the rather small sample size of our study population as well as for moderate renal insufficiency of our patients, which was not in advanced stage of CKD. In this study, we found that as obestatin levels rises, BCM and FFMI levels drop. These findings showed that obestatin might affect PEM and growth retardation indirectly due to its anorexigenic effects that influenced the body composition of CKD patients and consequently decreased the BCM and FFMI.

Our study had some limitations, including the small sample size that had a negative impact on the correlation analysis. In addition, this study was done only on predialysis patients and indeed, it was better to compare children with different stages of CKD. Moreover, in our study only the total ghrelin levels were measured, but for a better accuracy, it would have been better to measure the total- and acyl- ghrelin levels as well as their ratio. 

## Conclusion

Although in this study we could not observe significant changes in some appetite regulatory hormones, but such insignificant alteration of these three hormones in combination with other metabolic disorders such as uremia might influence and aggravate the protein-energy malnutrition and consequently its other complications. Further thorough studies are needed to clarify the role of appetite-regulatory hormones and their exact pathogenetic mechanisms in the etiology of CKD-associated protein energy malnutrition. 
